# Adult patients with Ph+ ALL benefit from conditioning regimen of medium‐dose VP16 plus CY/TBI

**DOI:** 10.1002/hon.3046

**Published:** 2022-07-10

**Authors:** Mari Morita‐Fujita, Yasuyuki Arai, Tadakazu Kondo, Kaito Harada, Naoyuki Uchida, Takashi Toya, Yukiyasu Ozawa, Takahiro Fukuda, Shuichi Ota, Makoto Onizuka, Yoshinobu Kanda, Yumiko Maruyama, Satoru Takada, Toshiro Kawakita, Takahide Ara, Tatsuo Ichinohe, Takafumi Kimura, Yoshiko Atsuta, Shinichi Kako

**Affiliations:** ^1^ Department of Hematology and Oncology Graduate School of Medicine Kyoto University Kyoto Japan; ^2^ Department of Clinical Laboratory Medicine Graduate School of Medicine Kyoto University Kyoto Japan; ^3^ Department of Hematology and Oncology Tokai University School of Medicine Isehara Japan; ^4^ Department of Hematology Toranomon Hospital Tokyo Japan; ^5^ Hematology Division Tokyo Metropolitan Cancer and Infectious Diseases Center Komagome Hospital Tokyo Japan; ^6^ Department of Hematology Japanese Red Cross Nagoya First Hospital Nagoya Japan; ^7^ Department of Hematopoietic Stem Cell Transplantation National Cancer Center Hospital Tokyo Japan; ^8^ Department of Hematology Sapporo Hokuyu Hospital Sapporo Japan; ^9^ Division of Hematology Jichi Medical University Saitama Medical Center Saitama Japan; ^10^ Department of Hematology University of Tsukuba Hospital Ibaraki Japan; ^11^ Leukemia Research Center Saiseikai Maebashi Hospital Maebashi Japan; ^12^ Department of Hematology National Hospital Organization Kumamoto Medical Center Kumamoto Japan; ^13^ Department of Hematology Hokkaido University Hospital Sapporo Japan; ^14^ Department of Hematology and Oncology Research Institute for Radiation Biology and Medicine Hiroshima University Hiroshima Japan; ^15^ Preparation Department Japanese Red Cross Kinki Block Blood Center Osaka Japan; ^16^ Japanese Data Center for Hematopoietic Cell Transplantation Nagakute Japan; ^17^ Department of Registry Science for Transplant and Cellular Therapy Aichi Medical University School of Medicine Nagakute Japan

**Keywords:** acute lymphoblastic leukemia, Philadelphia chromosome, VP16/CY/TBI

## Abstract

The medium‐dose etoposide (VP16) added on cyclophosphamide (CY)/total body irradiation (TBI) is one of the intensified myeloablative conditioning regimens used in allogenic hematopoietic stem cell transplantation (allo‐HSCT) for acute lymphoblastic leukemia (ALL). However, the patient subgroups who can actually benefit from VP16/CY/TBI compared to CY/TBI have not been precisely defined. Therefore, we conducted a multi‐center retrospective study using the Japanese nationwide registry database to elucidate the efficacy of VP16/CY/TBI on post‐transplant prognosis. Biological and clinical distinct subtypes (i.e., Philadelphia chromosome‐positive (Ph+) and ‐negative (Ph−) ALL) were evaluated separately, which included 820 Ph+ and 1463 patients with Ph− ALL, respectively. Compared with the CY/TBI group, the VP16/CY/TBI group showed superior progression‐free survival (PFS) in patients with Ph+ ALL (65% vs. 57% at 3 years after HSCT; adjusted hazard ratio (HR), 0.73; 95% confidence interval (CI), 0.55–0.98; *p* = 0.03), along with significantly reduced incidence of relapse (adjusted HR, 0.58; 95% CI, 0.37–0.90; *p* = 0.02) without the increase of non‐relapse mortality (NRM). By contrast, in patients with Ph− ALL, VP16/CY/TBI did not improve PFS nor incidence of relapse; addition of VP16 reduced relapse (HR, 0.65; *p* = 0.06) in patients with Ph− ALL transplanted at CR1, while improved PFS was not observed (HR, 0.90; *p* = 0.52) due to increased NRM. This study demonstrated that VP16/CY/TBI is a more effective and well‐tolerated regimen in comparison with CY/TBI in patients with myeloablative allo‐HSCT for adult Ph+ ALL. Our findings can provide a novel algorithm for conditioning regimen selection in patients with adult ALL.

## 1 INTRODUCTION

Intensified myeloablative conditioning (MAC) regimens in allogenic hematopoietic stem cell transplantation (allo‐HSCT) have long been used for the treatment of highly aggressive or refractory leukemia or lymphoma.^1^, ^2^, ^3^ Among these intensified MAC regimens, etoposide (VP16) added on cyclophosphamide (CY) and total body irradiation (TBI) is in the selection list of conditioning regimens for high‐risk acute lymphoblastic leukemia (ALL).^1^, ^4^, ^5^, ^6^ Although VP16/CY/TBI is effective in reducing post‐transplant relapse, regimen‐related toxicities have been the barrier for the wider application.^7^, ^8^ In recent years, several studies have reported that medium‐dose VP16 plus CY/TBI (VP16/CY/TBI; total dose of VP16, 30–40 mg/kg in comparison with 40–60 mg/kg in the conventional dose) is well‐tolerated and also useful in disease control as an intensified MAC regimen for adult ALL,^9^, ^10^, ^11^, ^12^ although these results are all from small and single‐arm studies. In our previous study analyzing patients who received bone marrow and peripheral blood stem cell transplantation (BMT/PBSCT) for ALL in complete remission (CR), we showed the superiority of VP16/CY/TBI to CY/TBI in the high‐risk population.^9^ However, further studies are still necessary to precisely define the patient subgroups who can actually benefit from VP16/CY/TBI.

Therefore, we conducted a multicenter retrospective study using a Japanese nationwide registry database to elucidate the influence of VP16 added to CY/TBI on post‐transplant prognosis. The biologically and clinically distinct groups (i.e., Philadelphia chromosome‐positive (Ph+) and ‐negative (Ph−) ALL) were analyzed separately regarding the efficacy and adverse events of VP16/CY/TBI in comparison with CY/TBI. Our findings can provide a novel strategy for conditioning regimen selection before allo‐HSCT for patients with adult ALL.

## 2 PATIENTS AND METHODS

### 2.1 Inclusion criteria

Data of patients with adult ALL (age ≥16 years) who underwent their first allogenic HSCT between January 2000 and December 2018 were obtained through the Japanese Transplant Registry Unified Management Program (TRUMP), sponsored by the Japanese Society for Transplantation and Cellular Therapy (JSTCT) and the Japanese Data Center for Hematopoietic Cell Transplantation (JDCHCT).^13^, ^14^ We included patients who received the following MAC or intensified MAC regimens: (1) CY/TBI (CY, total 120 mg/kg; TBI, 10–12 Gy), and (2) VP16/CY/TBI (VP16, total 30–40 mg/kg or approximately 1000 mg/m^2^). Pre‐transplant chemotherapies and transplant procedures including the choice of conditioning regimens and dosage of VP16 in case of the VP16/CY/TBI regimen were determined by each attending physician.

The study protocol complied with the standards outlined in the Helsinki Declaration and was approved by the Ethical Committee in Kyoto University (R1507). Personal information is anonymized, and written informed consent was obtained from each patient.

### 2.2 Data collection and definition of each covariate

From the TRUMP database, we extracted data on basic pre‐transplant characteristics and post‐transplant clinical courses. The total white blood cell counts at diagnosis >30 × 10^9^/L was considered as high risk and others as standard risk. Patients were categorized into two groups: older patients (age >35 years) and young patients (aged ≤35 years). Molecular response was mainly assessed by polymerase chain reaction (PCR)‐based amplification of BCR‐ABL1 for Ph+ ALL.^15^ The definition of minimal residual disease (MRD) for Ph− ALL is detection of chimeric mRNA or Ig/TCR rearrangement by PCR or leukemic cells by flowcytometry. In related (Rel) BMT/PBSCT, disparities of human leukocyte antigen (HLA) were assessed by serological matching in six loci (HLA‐A, B, and DR). In unrelated (unrelated (UR)) BMT/PBSCT, matching in eight locus including HLA‐C were assessed at allele level; a 6/6 (Rel) and 8/8 (UR) match was considered HLA‐matched. In UR‐CBT, grouping by HLA match/mismatch status was not performed because most patients received grafts from serologically HLA‐mismatched donors and influence of HLA mismatch on transplant prognosis have not been well established particularly in the adult population.^16^

Posttransplant clinical courses were collected and evaluated as previously described.^9^ Relapse was defined at the hematological level (morphological evidence of blast cells in bone marrow or extramedullary lesion). Engraftment of neutrophils and platelets was defined as the first day of 3 consecutive days during which neutrophil and platelet counts were at least 500/µL and 20 × 10^9^/L (without transfusion support), respectively. Diagnosis and classification of acute GVHD and chronic GVHD were performed by each attending physician based on conventional criteria.^17^, ^18^ Infection with bacteria, fungi such as *Candida* and *Aspergillus* spp., or viruses such as cytomegalovirus was diagnosed based on culture, serological test, and PCR as well as imaging findings. The cumulative incidences of infection were compared between the CY/TBI and VP16/CY/TBI groups.

### 2.3 Statistical analyses

Ph+ and Ph− ALL were separately evaluated in all analyses. Patients' characteristics and the cause of non‐relapse mortality (NRM) were compared between the groups using Fisher's exact tests for categorical variables and the Mann–Whitney U tests for continuous variables. For survival analysis, progression‐free survival (PFS) was measured from the date of HSCT to the last follow‐up or death from any cause or relapse; survival curves were described using the Kaplan–Meier method, and the groups were compared using the log‐rank test. Cumulative incidence curves for NRM and relapse were compared using the Gray test treating the relapse and NRM as a competing risk, respectively. In addition to analyses including a total cohort, PFS and cumulative incidence of NRM and relapse were also assessed according to the types of HSCT (BMT/PBSCT and CBT). To assess the effect of prognostic factors, we used the Cox proportional hazards regression model and the Fine–Gray proportional hazards model. Multivariate analyses were performed using the forced entry method including variables with significance or borderline significance in the univariate analyses along with the basic clinical characteristics either in the subgroup of Ph+ or Ph−. All statistical analyses were performed using R statistical software, version 3.6.1 (R Foundation for Statistical Computing, Vienna, Austria). All *p*‐values are two‐sided, and *p* < 0.05 are considered statistically significant.

## 3 RESULTS

### 3.1 Patient characteristics

A total of 2283 patients, aged 16–65 (median, 37) years were analyzed. The numbers of patients with Ph+ and Ph− ALL were 820 and 1463, respectively. The patient and transplant characteristics are shown in Table 1. The median follow‐up period for survivors was 51.3 (range, 0.4–217.0) months. In patients with Ph+ ALL, the VP16/CY/TBI group is associated with younger age, advanced disease status, and more recent HSCT than the CY/TBI group. In patients with Ph− ALL, a skewed distribution was observed in patient sex, donor type, sex disparity, and GVHD prophylaxis in addition to the above‐mentioned three variables (Table 1).

**TABLE 1:** Patient characteristics

		Ph‐positive					Ph‐negative				
		CY/TBI (*N* = 654)		VP16/CY/TBI (*N* = 166)			CY/TBI (*N* = 1054)		VP16/CY/TBI (*N* = 409)		
Variables		N	%	N	%	*p*	N	%	N	%	*p*
Patient age, y	Median (range)	41 (16–61)		37.5 (17–56)		<0.01*	36 (16–65)		32 (16–61)		<0.01*
	16–35	215	32.9	68	41.0	0.06	520	49.3	255	62.3	<0.01*
	36‐	439	67.1	98	59.0		534	50.7	154	37.7	
Patient sex	Female	276	42.2	68	41.0	0.79	478	45.4	157	38.4	0.02*
	Male	378	57.8	98	59.0		576	54.6	252	61.6	
HCT‐CI	0	434	66.4	107	64.5	0.20	682	64.7	284	69.4	0.33
	1‐	166	25.4	53	31.9		301	28.6	110	26.9	
Disease status	CR1	583	89.1	131	78.9	<0.01*	740	70.2	224	54.8	<0.01*
	CR2‐	25	3.8	13	7.8		145	13.8	64	15.6	
	NR	46	7.0	22	13.3		169	16.0	121	29.6	
MRD‐negative	Yes	418	63.9	96	57.8	0.04*	196	18.6	57	13.9	<0.01*
	No	201	30.7	67	40.3		286	27.1	165	40.3	
	Unknown	35	5.4	3	1.8		572	54.3	187	45.7	
Initial WBC	<30,000	324	49.5	88	53.0	0.43	708	67.2	256	62.6	0.07
	≥30,000	322	49.2	76	45.8		330	31.3	149	36.4	
Stem cell source	BM	425	65.0	103	62.0	0.59	665	63.1	230	56.2	<0.01*
	PBSC	135	20.6	34	20.5		231	21.9	84	20.5	
	CB	94	14.4	29	17.5		158	15.0	95	23.2	
Donor type	M‐RD	200	30.6	53	31.9	0.83	317	30.1	101	24.7	<0.01*
	MM‐RD	16	2.4	3	1.8		50	4.7	17	4.2	
	MUD	181	27.7	41	24.7		283	26.9	92	22.5	
	MMUD	153	23.4	40	24.1		219	20.8	98	24.0	
	UR‐CB	94	14.4	29	17.5		158	15.0	95	23.2	
Sex mismatch	Matched	329	50.3	83	50.0	0.86	563	53.4	207	50.6	0.02*
	M to F	154	23.5	37	22.3		270	25.6	85	20.8	
	F to M	153	23.4	42	25.3		189	17.9	97	23.7	
ABO disparity	Matched	331	50.6	66	39.8	0.04*	522	49.5	195	47.7	0.66
	Minor mismatch	133	20.3	41	24.7		216	20.5	94	23.0	
	Major mismatch	123	18.8	44	26.5		205	19.4	75	18.3	
	Both	62	9.5	15	9.0		105	10.0	45	11.0	
GVHD prophylaxis	CyA‐based	276	42.2	59	35.5	0.13	445	42.2	146	35.7	0.03*
	TAC‐based	369	56.4	105	63.3		595	56.5	254	62.1	
Year of HSCT	−2009	198	30.3	26	15.7	<0.01*	314	29.8	66	16.1	<0.01*
	2010‐	456	69.7	140	84.3		740	70.2	343	83.9	
TKI before HSCT	Yes	600	91.7	153	92.1	0.37					
	IMA	318	48.6	62	37.3						
	DASA	257	39.3	81	48.7						
	No	22	3.4	8	4.8						
TKI maintenance	Yes	40	6.1	11	6.6	0.86					
	No	116	17.7	17	10.2						
	Unknown	498	76.1	138	83.1						
VP16 dose, mg/kg^a^	Median (range)			30 (29–40)					30 (26–40)		
Follow‐up period, m	Median (range)	49.7 (1.9–194.9)		51.8 (0.4–136.8)		0.83	52.5 (0.4–217.0)		47.7 (1.7–211.4)		0.17

Abbreviations: BM, bone marrow; CR, complete remission; CB, cord blood; CyA, cyclosporine; DASA, dasatinib; GVHD, graft‐versus‐host disease; HCT‐CI, hematopoietic cell transplantation‐specific comorbidity index; HSCT, hematopoietic stem cell transplantation; IMA, imatinib; M‐RD, matched related donor; MM‐RD, mismatched related donor; MUD, matched unrelated donor; MMUD, mismatched unrelated donor; NCR, non remission; PBSC, peripheral blood stem cells; Tac, tacrolimus; TKI, tyrosine‐kinase inhibitor; UR‐CB, unrelated cord blood; WBC, white blood cell.

^a^ DuBois formula was used to convert dose per BSA (mg/m2) to dose per weight (mg/kg).

* indicates statistically significant, *p* < 0.05.

### 3.2 Improved prognosis with VP16/CY/TBI in patients with Ph+ ALL

**FIGURE 1:**
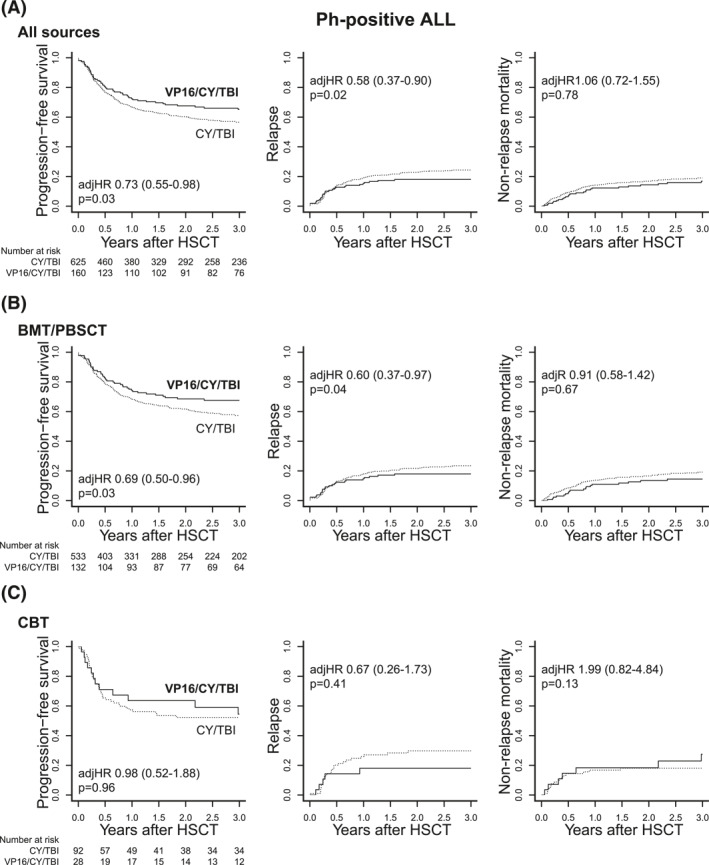
Comparison of post‐HSCT prognosis in patients with Ph+ acute lymphoblastic leukemia (ALL) according to the conditioning regimen. Comparison of progression‐free survival (PFS), relapse, and non‐relapse mortality (NRM) between the cyclophosphamide (CY)/total body irradiation (TBI) and VP16/CY/TBI group (A) in a whole cohort of patients with Ph+ ALL, patients with Ph+ ALL treated with (B) BMT/PBSCT and (C) CBT. VP16/CY/TBI cohort is shown in thick lines, while CY/TBI in dot lines

We evaluated the effect of VP16 addition on the outcomes after HSCT in patients with Ph+ ALL. The VP16/CY/TBI group showed higher PFS in the whole cohort of patients with Ph+ ALL (65% vs. 57% at 3 years after HSCT; adjusted hazard ratio (HR), 0.73; 95% confidence interval (CI), 0.55–0.98; *p* = 0.03) (Figure 1A and Table 2). This prognostic difference between VP16/CY/TBI and CY/TBI was more apparent in HSCTs from BMT/PBSCT donors than from CBT (adjusted HR, 0.69; 95% CI, 0.50–0.96; *p* = 0.03 for BMT/PBSCT vs. HR, 0.98; 95% CI, 0.52–1.88; *p* = 0.96 for CBT) (Figure 1B,C). No significant difference in OS was seen between the VP16/CY/TBI and CY/TBI groups in the whole cohort of patients with Ph+ ALL (71% vs. 66% at 3 years after HSCT).

**TABLE 2:** Uni‐ and multi‐variate analyses for progression‐free survival (PFS), relapse, and non‐relapse mortality (NRM) in Ph+ patients

		Progression‐free survival				Relapse				Non‐relapse mortality			
		Univariate		Multivariate		Univariate		Multivariate		Univariate		Multivariate	
Variables		HR (95% CI)	*p*	HR (95% CI)	*p*	HR (95% CI)	*p*	HR (95% CI)	*p*	HR (95% CI)	*p*	HR (95% CI)	*p*
Conditioning	CY/TBI	1.00 (ref)				1.00 (ref)				1.00 (ref)			
	VP16/CY/TBI	0.83 (0.63–1.1)	0.19	0.73 (0.55–0.98)	0.03*	0.74 (0.50–1.11)	0.15	0.58 (0.37–0.90)	0.02*	1.01 (0.69–1.47)	0.96	1.06 (0.72–1.55)	0.78
Patient age	16–35	1.00 (ref)											
	36‐	1.02 (0.81–1.27)	0.90	1.07 (0.84–1.35)	0.59	0.75 (0.56–1.01)	0.06	0.73 (0.53–0.99)	0.05*	1.49 (1.04–2.14)	0.03*	1.61 (1.10–2.36)	0.02*
Patient sex	Female	1.00 (ref)				1.00 (ref)				1.00 (ref)			
	Male	1.04 (0.84–1.30)	0.70	1.01 (0.81–1.27)	0.92	0.93 (0.69–1.26)	0.65	0.93 (0.68–1.27)	0.63	1.20 (0.87–1.67)	0.26	1.19 (0.85–1.67)	0.31
HCT‐CI	0	1.00 (ref)				1.00 (ref)				1.00 (ref)			
	1‐	1.19 (0.93–1.53)	0.16	1.16 (0.91–1.5)	0.24	1.16 (0.83–1.63)	0.38	1.16 (0.81–1.67)	0.42	1.14 (0.80–1.63)	0.48	1.10 (0.76–1.58)	0.62
Disease status	CR1	1.00 (ref)				1.00 (ref)				1.00 (ref)			
	CR2‐	3.38 (2.30–4.98)	<0.01*	3.35 (2.22–5.05)	<0.01*	2.92 (1.72–4.98)	<0.01*	2.90 (1.66–5.04)	<0.01*	2.10 (1.16–3.79)	0.01*	2.08 (1.12–3.86)	0.02*
	NR	3.79 (2.80–5.12)	<0.01*	3.87 (2.82–5.29)	<0.01*	4.92 (3.35–7.23)	<0.01*	5.10 (3.37–7.71)	<0.01*	1.03 (0.58–1.83)	0.92	1.01 (0.56–1.81)	0.99
MRD‐negative	Yes	1.00 (ref)				1.00 (ref)				1.00 (ref)			
	No	2.26 (1.81–2.83)	<0.01*			3.03 (2.22–4.11)	<0.01*			1.18 (0.85–1.65)	0.32		
Initial WBC	<30,000	1.00 (ref)				1.00 (ref)				1.00 (ref)			
	≥30,000	1.33 (1.08–1.66)	<0.01*	1.14 (0.91–1.43)	0.24	1.67 (1.24–2.26)	<0.01*	1.34 (0.97–1.85)	0.08	0.91 (0.66–1.24)	0.54	0.86 (0.62–1.20)	0.38
Donor type	M‐RD	1.00 (ref)				1.00 (ref)				1.00 (ref)			
	MM‐RD	1.24 (0.60–2.55)	0.56	0.98 (0.47–2.06)	0.96	0.90 (0.32–2.48)	0.83	0.50 (0.14–1.79)	0.29	1.55 (0.53–4.50)	0.42	1.53 (0.52–4.49)	0.44
	MUD	1.03 (0.77–1.37)	0.86	0.95 (0.67–1.34)	0.77	0.98 (0.68–1.43)	0.94	1.08 (0.67–1.74)	0.74	1.06 (0.68–1.67)	0.79	0.87 (0.51–1.47)	0.60
	MMUD	1.00 (0.74–1.35)	0.99	0.91 (0.63–1.31)	0.60	0.61 (0.39–0.95)	0.03*	0.69 (0.40–1.18)	0.18	1.60 (1.05–2.46)	0.03*	1.26 (0.76–2.09)	0.37
	UR‐CB	1.39 (1.01–1.92)	0.04*	1.36 (0.95–1.95)	0.10	1.10 (0.71–1.70)	0.68	1.20 (0.73–1.99)	0.47	1.60 (0.99–2.58)	0.06	1.32 (0.77–2.25)	0.31
Sex mismatch	Matched	1.00 (ref)				1.00 (ref)				1.00 (ref)			
	M to F	1.12 (0.86–1.47)	0.40			1.05 (0.74–1.51)	0.78			1.12 (0.74–1.70)	0.58		
	F to M	1.01 (0.77–1.32)	0.95			0.78 (0.53–1.13)	0.19			1.37 (0.94–1.98)	0.10		
ABO mismatch	Matched	1.00 (ref)				1.00 (ref)				1.00 (ref)			
	Minor mis	1.01 (0.76–1.34)	0.93			0.78 (0.52–1.18)	0.24			1.35 (0.92–1.97)	0.13		
	Major mis	1.12 (0.84–1.49)	0.43			1.04 (0.7–1.53)	0.85			1.13 (0.74–1.73)	0.57		
	Both	1.41 (0.98–2.02)	0.06			1.55 (0.97–2.48)	0.07			1.01 (0.56–1.83)	0.97		
GVHD prophylaxis	CyA‐based	1.00 (ref)				1.00 (ref)				1.00 (ref)			
	TAC‐based	0.93 (0.75–1.16)	0.53	1.08 (0.81–1.45)	0.59	0.69 (0.51–0.93)	0.01*	0.81 (0.54–1.21)	0.30	1.31 (0.95–1.81)	0.10	1.40 (0.91–2.15)	0.13
Year of HSCT	−2009	1.00 (ref)				1.00 (ref)				1.00 (ref)			
	2010‐	0.64 (0.51–0.80)	<0.01*	0.75 (0.57–0.97)	0.03*	0.69 (0.51–0.93)	0.02*	1.02 (0.69–1.53)	0.91	0.66 (0.47–0.92)	0.01*	0.59 (0.41–0.87)	<0.01*

*Note*: Other abbreviations are shown in Table 1.

Abbreviations: CI; confidence interval; HR, hazard ratio.

As one of the reasons of superior PFS in the VP16/CY/TBI group, significantly reduced incidence of relapse was observed in this group as a whole (adjusted HR, 0.58; 95% CI, 0.37–0.90; *p* = 0.02) (Figure 1A and Table 2) or in the BMT/PBSCT group (adjusted HR, 0.60; 95% CI, 0.37–0.97; *p* = 0.04) (Figure 1B). In CBT, however, reduction in relapse was not significant (adjusted HR, 0.67; 95% CI, 0.26–1.73; *p* = 0.41) (Figure 1C and Table 2). The percentage of patients on whom post‐transplant tyrosine kinase inhibitors (TKI) maintenance therapy was used showed no significant difference between VP16/CY/TBI and CY/TBI groups.

As for NRM, no significant difference was detected between the VP16/CY/TBI group and the CY/TBI group in the total cohort (adjusted HR, 1.06; 95% CI, 0.72–1.55; *p* = 0.78) (Figure 1A and Table 2) or in each group of BMT/PBSCT and CBT (Figure 1B,C).

**FIGURE 2:**
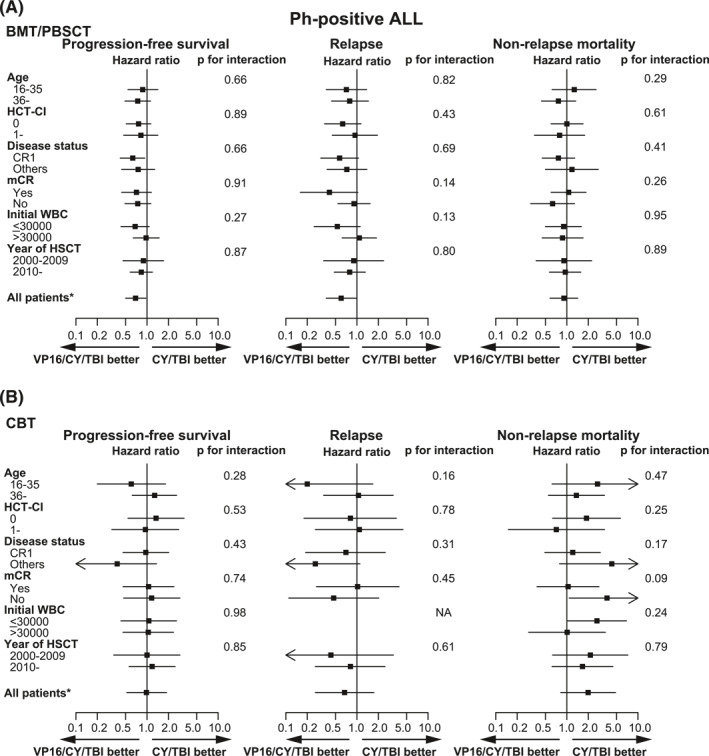
Subgroup analyses of post‐HSCT outcomes in patients with Ph+ acute lymphoblastic leukemia (ALL). Progression‐free survival (PFS), relapse, and non‐relapse mortality (NRM) were analyzed in detail by each subgroup with respect to patient characteristics for patients with Ph+ ALL who received (A) BMT/PBSCT and (B) CBT. HRs are shown by black boxes, with the lines indicating 95% confidence intervals. *p* values were obtained from the interaction test between the conditioning regimen and patient characteristics

Now that we recognized the prognostic difference between BMT/PBSCT and CBT groups in patients with Ph+ ALL, we performed separate subgroup analyses in each donor group (Figure 2). As a result, no significant interactions were found between the effect of additional VP16 and PFS, relapse, or NRM (Figure 2A,B). The positive effect of VP16/CY/TBI on PFS and relapse was more clearly observed among subgroups in BMT/PBSCT (Figure 2A) than in CBT (Figure 2B).

To assess whether addition of VP16 can increase post‐HSCT toxicities, engraftment and incidence of GVHD and infection were compared between the two groups (Supplemental Figure 1). Analyses started from the BMT/PBSCT cohort, and the incidence of engraftment including the period before engraftment was comparable irrespective of VP16 addition (Supplemental Figure 1A). Acute and chronic GVHD were also observed in the same frequencies without any significant differences (Supplemental Figure 1B and C). By contrast, the incidence of bacterial and viral infections showed the tendency to increase in the VP16/CY/TBI group (Supplemental Figure 1D). Analyses in the CBT cohort showed the same results as those in the BMT/PBSCT cohort (Supplemental Figure 1E–G), except for the absence of difference in the incidence of infection between the two groups of VP16/CY/TBI and CY/TBI (Supplemental Figure 1H). In addition to these toxicities, causes of NRM were also analyzed (Supplemental Table 1). No specific causes of NRM were correlated with the VP16/CY/TBI group.

### 3.3 No survival advantage with VP16/CY/TBI observed in patients with Ph− ALL

**FIGURE 3:**
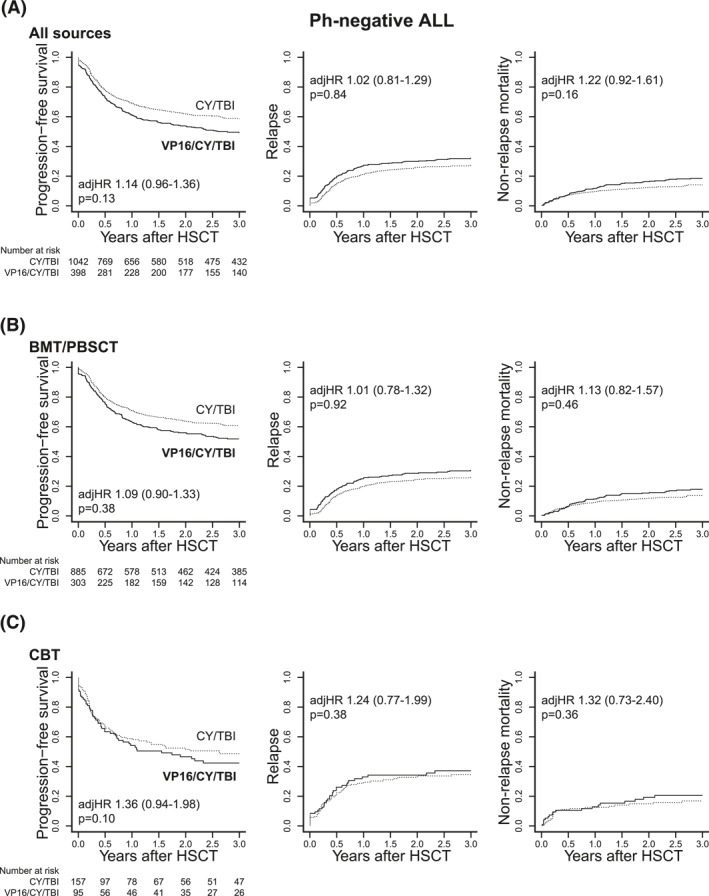
Comparison of post‐HSCT prognosis in patients with Ph− acute lymphoblastic leukemia (ALL) according to the conditioning regimen. Comparison of progression‐free survival (PFS), relapse, and non‐relapse mortality (NRM) between the cyclophosphamide (CY)/total body irradiation (TBI) and VP16/CY/TBI group (A) in a whole cohort of patients with Ph− ALL, Patients with Ph− ALL treated with (B) BMT/PBSCT and (C) CBT. VP16/CY/TBI cohort is shown in thick lines, while CY/TBI in dot lines

In contrast to the significant improvement in the prognosis with VP16/CY/TBI among patients with Ph+ ALL treated with BMT/PBSCT, VP16/CY/TBI did not improve PFS in the total cohort of patients with Ph− ALL (adjusted HR, 1.14; 95% CI, 0.96–1.36; *p* = 0.13) (Figure 3A and Table 3). The results were consistent in the subgroup analyses for donor sources (adjusted HR, 1.09; 95% CI, 0.90–1.33; *p* = 0.38 for BMT/PBSCT; adjusted HR, 1.36; 95% CI, 0.94–1.98; *p* = 0.10 for CBT) (Figure 3B,C). Addition of VP16 did not have any influence on disease relapse for the total cohort (adjusted HR, 1.02; 95% CI, 0.81–1.29; *p* = 0.84) (Figure 3A and Table 3) and for each group of donor source (Figure 3B,C). NRM was slightly inferior in the VP16/CY/TBI group (Figure 3B,C and Table 3). OS was also similar between the VP16/CY/TBI and CY/TBI groups in the whole cohort of patients with Ph− ALL (56% vs. 63% at 3 years after HSCT).

**TABLE 3:** Uni‐ and multi‐variate analyses for progression‐free survival (PFS), relapse, and non‐relapse mortality (NRM) in Ph− patients

		Progression‐free survival				Relapse				Non‐relapse mortality			
		Univariate		Multivariate		Univariate		Multivariate		Univariate		Multivariate	
Variables		HR (95% CI)	*p*	HR (95% CI)	*p*	HR (95% CI)	*p*	HR (95% CI)	*p*	HR (95% CI)	*p*	HR (95% CI)	*p*
Conditioning	CY/TBI	1.00 (ref)				1.00 (ref)				1.00 (ref)			
	VP16/CY/TBI	1.37 (1.16–1.61)	<0.01*	1.14 (0.96–1.36)	0.13	1.24 (1.00–1.53)	0.05	1.02 (0.81–1.29)	0.84	1.40 (1.08–1.81)	0.01*	1.22 (0.92–1.61)	0.16
Patient age	16–35	1.00 (ref)				1.00 (ref)				1.00 (ref)			
	36‐	1.05 (0.90–1.23)	0.52	1.29 (1.10–1.51)	<0.01*	0.91 (0.75–1.11)	0.38	1.07 (0.86–1.32)	0.54	1.23 (0.96–1.57)	0.10	1.38 (1.06–1.78)	0.02*
Patient sex	Female	1.00 (ref)				1.00 (ref)				1.00 (ref)			
	Male	1.21 (1.03–1.41)	0.02*	1.23 (1.05–1.44)	0.01*	0.99 (0.81–1.21)	0.93	0.96 (0.78–1.18)	0.67	1.50 (1.16–1.94)	<0.01*	1.56 (1.20–2.02)	<0.01*
HCT‐CI	0	1.00 (ref)				1.00 (ref)				1.00 (ref)			
	1‐	1.24 (1.05–1.47)	0.01*	1.15 (0.97–1.37)	0.11	1.20 (0.97–1.49)	0.10	1.09 (0.87–1.37)	0.45	1.19 (0.91–1.57)	0.21	1.08 (0.82–1.44)	0.58
Disease status	CR1	1.00 (ref)				1.00 (ref)				1.00 (ref)			
	CR2‐	2.06 (1.66–2.56)	<0.01*	2.14 (1.71–2.69)	<0.01*	1.80 (1.37–2.37)	<0.01*	1.93 (1.44–2.58)	<0.01*	1.91 (1.39–2.63)	<0.01*	1.89 (1.33–2.68)	<0.01*
	nCR	5.00 (4.20–5.95)	<0.01*	4.91 (4.09–5.89)	<0.01*	4.77 (3.83–5.94)	<0.01*	4.80 (3.81–6.05)	<0.01*	1.80 (1.34–2.43)	<0.01*	1.71 (1.26–2.34)	<0.01*
MRD‐negative	Yes	1.00 (ref)				1.00 (ref)				1.00 (ref)			
	No	3.73 (2.88–4.84)	<0.01*			4.39 (3.09–6.23)	<0.01*			1.50 (1.02–2.22)	0.04*		
Initial WBC	<30,000	1.00 (ref)				1.00 (ref)				1.00 (ref)			
	≥30,000	1.06 (0.90–1.25)	0.51	1.09 (0.92–1.29)	0.30	1.21 (0.98–1.49)	0.07	1.27 (1.02–1.59)	0.03*	0.80 (0.61–1.06)	0.12	0.80 (0.60–1.05)	0.11
Donor type	M‐RD	1.00 (ref)				1.00 (ref)				1.00 (ref)			
	MM‐RD	1.62 (1.16–2.27)	<0.01*	1.43 (1.00–2.04)	0.05*	1.35 (0.88–2.05)	0.17	0.96 (0.60–1.54)	0.86	1.83 (1.03–3.25)	0.04*	2.01 (1.08–3.73)	0.03*
	MUD	0.87 (0.70–1.09)	0.23	0.90 (0.70–1.15)	0.39	0.70 (0.53–0.91)	<0.01*	0.61 (0.44–0.84)	<0.01*	1.36 (0.95–1.96)	0.10	1.71 (1.11–2.64)	0.02*
	MMUD	0.97 (0.78–1.21)	0.80	0.91 (0.70–1.19)	0.49	0.62 (0.46–0.83)	<0.01*	0.48 (0.34–0.69)	<0.01*	1.89 (1.32–2.69)	<0.01*	2.38 (1.55–3.65)	<0.01*
	UR‐CB	1.46 (1.16–1.82)	<0.01*	1.13 (0.89–1.44)	0.32	1.13 (0.85–1.49)	0.40	0.75 (0.55–1.02)	0.07	1.77 (1.20–2.60)	<0.01*	1.82 (1.19–2.80)	<0.01*
Sex mismatch	Matched	1.00 (ref)				1.00 (ref)				1.00 (ref)			
	M to F	0.95 (0.78–1.15)	0.57			1.08 (0.85–1.37)	0.55			0.78 (0.56–1.07)	0.13		
	F to M	1.03 (0.85–1.27)	0.74			1.02 (0.78–1.32)	0.90			1.05 (0.77–1.43)	0.77		
ABO mismatch	Matched	1.00 (ref)				1.00 (ref)				1.00 (ref)			
	Minor mismatch	1.19 (0.97–1.45)	0.09			1.13 (0.88–1.45)	0.35			1.22 (0.89–1.68)	0.22		
	Major mismatch	1.28 (1.04–1.57)	0.02*			1.08 (0.83–1.41)	0.55			1.43 (1.03–1.97)	0.03*		
	Both	1.33 (1.03–1.72)	0.03*			1.20 (0.86–1.66)	0.29			1.37 (0.91–2.07)	0.13		
GVHD prophylaxis	CyA‐based	1.00 (ref)				1.00 (ref)				1.00 (ref)			
	TAC‐based	0.94 (0.80–1.10)	0.44	0.91 (0.75–1.11)	0.36	0.89 (0.73–1.08)	0.25	1.20 (0.93–1.55)	0.17	1.00 (0.78–1.29)	0.98	0.64 (0.46–0.88)	<0.01*
Year of HSCT	−2009	1.00 (ref)				1.00 (ref)				1.00 (ref)			
	2010‐	0.97 (0.82–1.16)	0.74	0.83 (0.68–1.01)	0.06	0.84 (0.68–1.04)	0.10	0.72 (0.57–0.92)	<0.01*	1.15 (0.87–1.52)	0.32	1.13 (0.81–1.58)	0.46

*Note*: Abbreviations are shown in Table 2.

**FIGURE 4:**
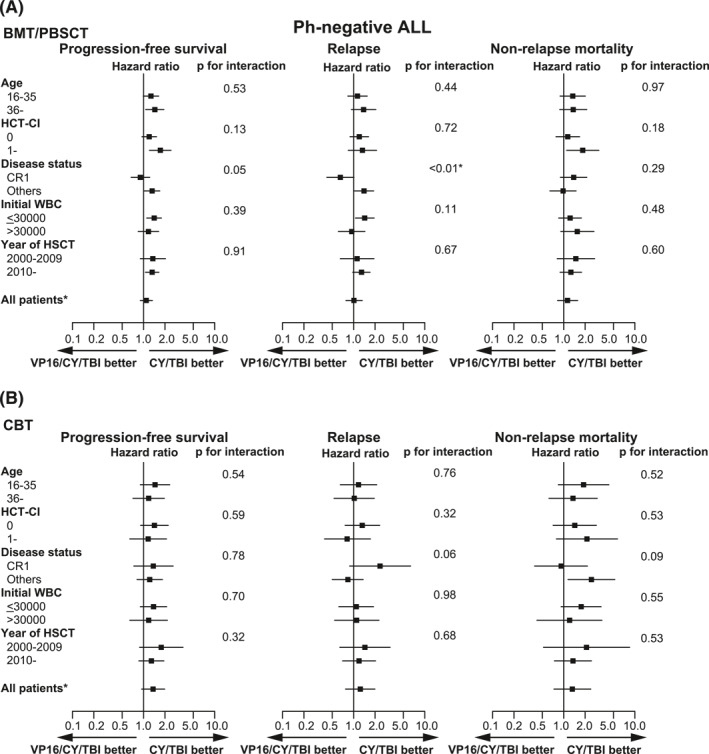
Subgroup analyses of post‐HSCT outcomes in patients with Ph‐ acute lymphoblastic leukemia (ALL). Progression‐free survival (PFS), relapse, and non‐relapse mortality (NRM) were analyzed in detail by each subgroup with respect to patient characteristics for patients with Ph− ALL who received (A) BMT/PBSCT and (B) CBT. HRs are shown by black boxes, with the lines indicating 95% confidence interval (CI). *p* values were obtained from interaction test between the conditioning regimen and patient characteristics

These results that VP16/CY/TBI did not improve PFS were confirmed in each subgroup (Figure 4). Of note, disease risk (CR1 vs. others) had significant interactions with VP16 usage with respect to PFS and relapse in patients treated with BMT/PBSCT (*p* for interaction, 0.05 and < 0.01, respectively), and addition of VP16 reduced relapse (HR, 0.65; 95% CI, 0.42–1.01; *p* = 0.06) in the patients at CR1, which was not observed in patients beyond CR1 (HR, 1.40; 95% CI, 1.03–1.91; *p* = 0.03) (Figure 4A). However, increased NRM offset this merit, and improved PFS was not observed in this subgroup (HR, 0.90; 95% CI, 0.67–1.22; *p* = 0.52).

Post‐transplant clinical courses were equivalent between VP16/CY/TBI and CY/TBI (Supplemental Figure 2) except for a significant increase in bacterial and viral infections in the VP16‐added group with BMT/PBSCT (Supplemental Figure 2D). Idiopathic pneumonitis and hemorrhage were more frequently observed as causes of NRM in the VP16/CY/TBI group (Supplemental Table 2).

## 4 DISCUSSION

This retrospective multicenter study using the nationwide registry database in Japan investigated the influence of additional medium‐dose VP16 on CY/TBI in patients with adult ALL and revealed two major findings: (1) VP16 improved PFS by suppressing relapse without increasing the TRM in patients with Ph+ ALL, and (2) addition of VP16 did not improve the PFS in patients with Ph− ALL as a whole or in any subgroups due to the increased incidence of NRM without attenuation of relapse.

We previously reported that VP16/CY/TBI improved PFS of patients with ALL in CR status with advanced risks including positive MRD, poor‐risk karyotypes, initial elevated leukocyte count, and HSCT at CR2 or later without including the subgroup analyses focusing exclusively on patients with Ph+ ALL.^9^ The present current study has changed the subgrouping methodology from the previous study, and for the first time, identified patients with Ph+ ALL as the subgroup where the addition of VP16 on CY/TBI is effective from the viewpoints of relapse, NRM, and PFS with statistical significance.

In patients with Ph+ ALL, additional effects of VP16 were observed in all patient subgroups in terms of the pre‐HSCT characteristics. We did not find any clinical features significantly interacting with the effects of VP16 on relapse. The interaction with MRD status and incidence of relapse was not observed (*p* for interaction, 0.14), and this result is consistent with previous studies that patients with Ph+ ALL conditioned with VP16/TBI‐based regimens showed favorable outcome, even though they included patients with MRD.^10^, ^19^ These data as a whole indicate that VP16/CY/TBI can be a recommended regimen for patients with Ph+ ALL if eligible for MAC regimens.

Moreover, the influence of VP16 added to CY/TBI showed quite a different tendency in patients with Ph− ALL than in patients with Ph+ ALL; NRM was significantly higher without significant attenuation of post‐transplant relapse, resulting in the similar or rather inferior PFS in VP16/CY/TBI than in CY/TBI. Higher incidence of NRM in VP16/CY/TBI subgroups may be related to more intensive history of prior chemotherapies, which are speculated from the higher percentage of patients without CR (29.6%) and longer duration from the diagnosis to HSCT (median, 209 days in Ph−, vs. 176 days in Ph+ , *p* < 0.01). Significantly enhanced NRM in patients with HCT‐CI ≥ 1 with BMT/PBSCT indicates that clinically apparent organ dysfunctions carried over from the preceding chemotherapies can enhance the toxicity of the conditioning regimen intensified with VP16. By contrast, absence in decrease of relapse with additional VP16 in this group is attributed to prior chemotherapy resistance, which is difficult to assume as well, albeit still reasonable. This speculation is supported by the findings that disease status was interacted with the effect of VP16/CY/TBI on relapse (Figure 4), and VP16/CY/TBI was associated with reduced relapse only for HSCT at CR1, but not associated with HSCT at other advanced stages in BMT/PBSCT (Figure 4). Considering that treatment of Ph− ALL mainly consists of multiple chemotherapeutic drugs,^20^, ^21^ most of the leukemic cells in advanced stages can no longer be susceptible for chemotherapies at the timing of HSCT^22^ even with the intensified MAC regimens.

These differences in prognosis between Ph+ ALL and Ph− ALL may be derived from the availability of TKI as leukemia‐targeted therapies as well as the pathophysiological features of leukemia cells. As patients with Ph+ ALL usually require chemotherapies with less intensity in combination with TKIs,^23^ adverse events (clinical or sub‐clinical) and resistance to chemotherapies during the induction, consolidation, and maintenance therapies, if required, may be significantly reduced in Ph+ ALL in comparison with Ph− ALL. Recently, in patients with Ph− ALL, several targeted therapies such as blinatumomab and inotuzumab ozogamicin are available,^24^, ^25^ and re‐evaluation will be necessary for patients with Ph− ALL who receive targeted therapies instead of conventional chemotherapies before allo‐HSCT.

This study thus investigated the influence of additional medium‐dose VP16 to CY/TBI in patients with Ph+ or Ph− adult ALL using the large real‐world database. However, this study has several limitations. For instance, owing to the retrospective nature, the selection of conditioning regimens (e.g., VP16/CY/TBI vs. CY/TBI) is at the discretion of the attending hematologists; this will cause the unmeasurable selection bias, even though outcomes are adjusted regarding the major patient characteristics using the multivariate analyses. This bias may be greater and affect the result especially in Ph− patients, given that the ratio of patients with poor disease control as well as undetermined MRD is higher in Ph− patients. In addition, detailed information on treatments before and after allo‐HSCT were not covered in the database; pediatric‐like regimens before allo‐HSCT are reportedly related to increased NRM,^26^ and TKI maintenance have been reported to improve PFS.^27^ Although TKI maintenance is an important confounding factor that affects relapse, the information was lacking in the majority of the patients and it was not evaluated in this study. Conclusions in this study should be validated after integrating data about TKI maintenance in future studies.

Intensified CY/TBI regimens were developed in the 1990s, but the problem was high NRM rate.^7^, ^8^ With improved management of post‐HSCT complications, the number of cases has increased in recent years, making it possible to compare the intensified regimens and standard CY/TBI. One of the other intensified regimens commonly used for ALL is high‐dose cytarabine (HDCA)/CY/TBI. No studies have evaluated the effect of HDCA/CY/TBI in Ph+ and Ph− groups respectively. Retrospective analysis of Japanese registry data showed that HDCA/CY/TBI improved prognosis in CBT, but had no effectiveness due to increased NRM in BMT/PBSCT.^28^, ^29^ In this study, the benefit of VP16/CY/TBI was less apparent in CBT than in BMT/PBSCT, although the number of cases was smaller in CBT. Given that HDCA/CY/TBI showed higher neutrophil engraftment rate than CY/TBI in CBT,^28^ HDCA/CY/TBI might have additional advantage besides relapse‐reducing effect and be more optimal than VP16/CY/TBI in CBT. The prospective comparative study is needed to answer this question.

In conclusion, we demonstrated that VP16/CY/TBI is more effective and well‐tolerated regimen in comparison with CY/TBI in myeloablative allo‐HSCT for patients with adult Ph+ ALL, and this effectiveness is observed only in a limited manner in patients with Ph− ALL. Further validation and comparison in prospective studies should be necessary to promote effective application of VP16/CY/TBI and to improve the prognosis in both patients with Ph+ and Ph− ALL. These data can be highly evaluated against the backdrop of an evolving landscape for ALL, that is, immunotherapies such as CAR‐T and blinatumomab as well as newer TKIs pre‐ and post‐HSCT.

## AUTHOR CONTRIBUTIONS

Mari Morita‐Fujita and Yasuyuki Arai designed the study, reviewed and analyzed data, and wrote the paper; Tadakazu Kondo, Kaito Harada, and Shinichi Kako interpreted data and revised the manuscript; Naoyuki Uchida, Takashi Toya, Yukiyasu Ozawa, Takahiro Fukuda, Shuichi Ota, Makoto Onizuka, Yoshinobu Kanda, Yumiko Maruyama, Satoru Takada, Toshiro Kawakita, Takahide Ara, Tatsuo Ichinohe, Takafumi Kimura, and Yoshiko Atsuta contributed to the data collection and provided critiques on the manuscript.

## CONFLICT OF INTEREST

The authors have no competing interests.

### PEER REVIEW

The peer review history for this article is available at https://publons.com/publon/10.1002/hon.3046.

## Supporting information

Supplementary Material S1

## Data Availability

The data that support the findings of this study are available on request from the corresponding author.
