# Supplementary material for: Adult patients with Ph+ ALL benefit from conditioning regimen of medium‐dose VP16 plus CY/TBI
Source: Hematol Oncol. 2022 Jul 10;40(5):1041–55. doi: 10.1002/hon.3046 (PMC10084153; doi:10.1002/hon.3046)

Supplemental Figure Legends

Supplemental Figure 1. Post-HSCT clinical courses after HSCT in patients with Ph+ ALL according to the conditioning regimen

The cumulative incidences of major events after HSCT such as neutrophil or platelet engraftment, acute and chronic GVHD, and infections were compared in patients with Ph+ ALL treated with (**A**-**D**) BMT/PBSCT and (**E**-**H**) CBT. VP16/CY/TBI cohort is shown in thick lines, while CY/TBI in dot lines. Abbreviations: Neut, neutrophil; Plt, platelet.

Supplemental Figure 2. Post-HSCT clinical courses after HSCT in patients with Ph− ALL according to the conditioning regimen

The cumulative incidences of major events after HSCT such as neutrophil or platelet engraftment, acute and chronic GVHD, and infections were compared in patients with Ph− ALL treated with (**A**-**D**) BMT/PBSCT and (**E**-**H**) CBT. The VP16/CY/TBI cohort is shown in thick lines, while CY/TBI in dot lines. Abbreviations: Neut, neutrophil; Plt, platelet.

Supplemental Tables

Supplemental Table1. Causes of non-relapse mortality in Ph+ patients

	CY/TBI (N = 654)		VP16/CY/TBI (N = 166)		p
	N	%	N	%	
Infection	45	6.9	11	6.6	1.00
bacterial	24		6		
fungal	7		2		
viral	7		0		
GVHD	14	2.1	6	3.6	0.27
acute	6		3		
chronic	8		3		
ARDS	6	0.9	1	0.6	1.00
IP	7	1.1	1	0.6	1.00
Hemorrhage	7	1.1	2	1.2	1.00
TMA/VOD	13	2.0	3	1.8	1.00
Organ failure	17	2.6	4	2.4	1.00
CNS	1		0		
heart	3		2		
lung	5		0		
kidney	6		1		
Graft failure	2	0.3	1	0.6	0.49
Secondary malignancy	3	0.5	1	0.6	1.00
Others	9	1.4	3	1.8	0.72
Total	123	18.8	33	19.9	0.74

Abbreviations: ARDS, acute respiratory distress syndrome; IP, interstitial pneumonia; TMA, thrombotic microangiopathy; VOD, veno-occlusive disease; and CNS, central nervous system. Other abbreviations are shown in Table 1.

Supplemental Table 2. Causes of non-relapse mortality in Ph− patients

	CY/TBI (N = 1054)		VP16/CY/TBI (N = 409)		p
	N	%	N	%	
Infection	39	3.7	17	4.2	0.65
bacterial	19		5		
fungal	4		8		
viral	9		3		
GVHD	23	2.2	9	2.2	1.00
acute	12		6		
chronic	11		3		
ARDS	6	0.6	3	0.7	0.72
IP	7	0.7	10	2.4	0.01*
Hemorrhage	5	0.5	7	1.7	0.05*
TMA/VOD	15	1.4	8	2.0	0.48
Organ failure	28	2.7	12	2.9	0.72
CNS	1		1		
heart	0		3		
lung	12		6		
kidney	6		1		
Graft failure	4	0.4	0	0.0	0.58
Secondary malignancy	3	0.3	4	1.0	0.10
Others	35	3.3	15	3.6	0.75
Total	165	15.7	85	20.8	0.02*

Abbreviations are shown in Supplemental Table 1.

Supplemental Figure 1

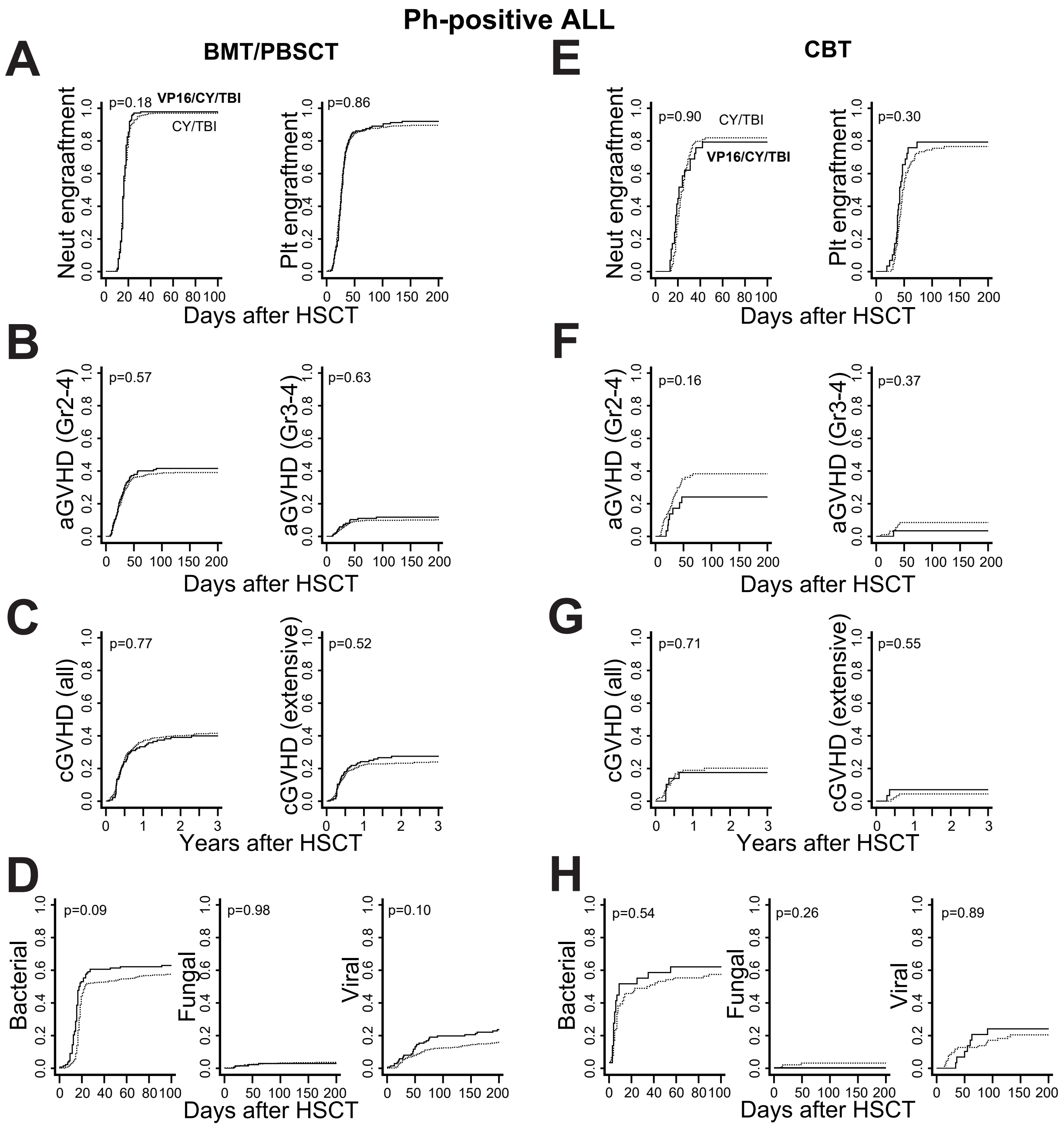

Supplemetal Figure 2

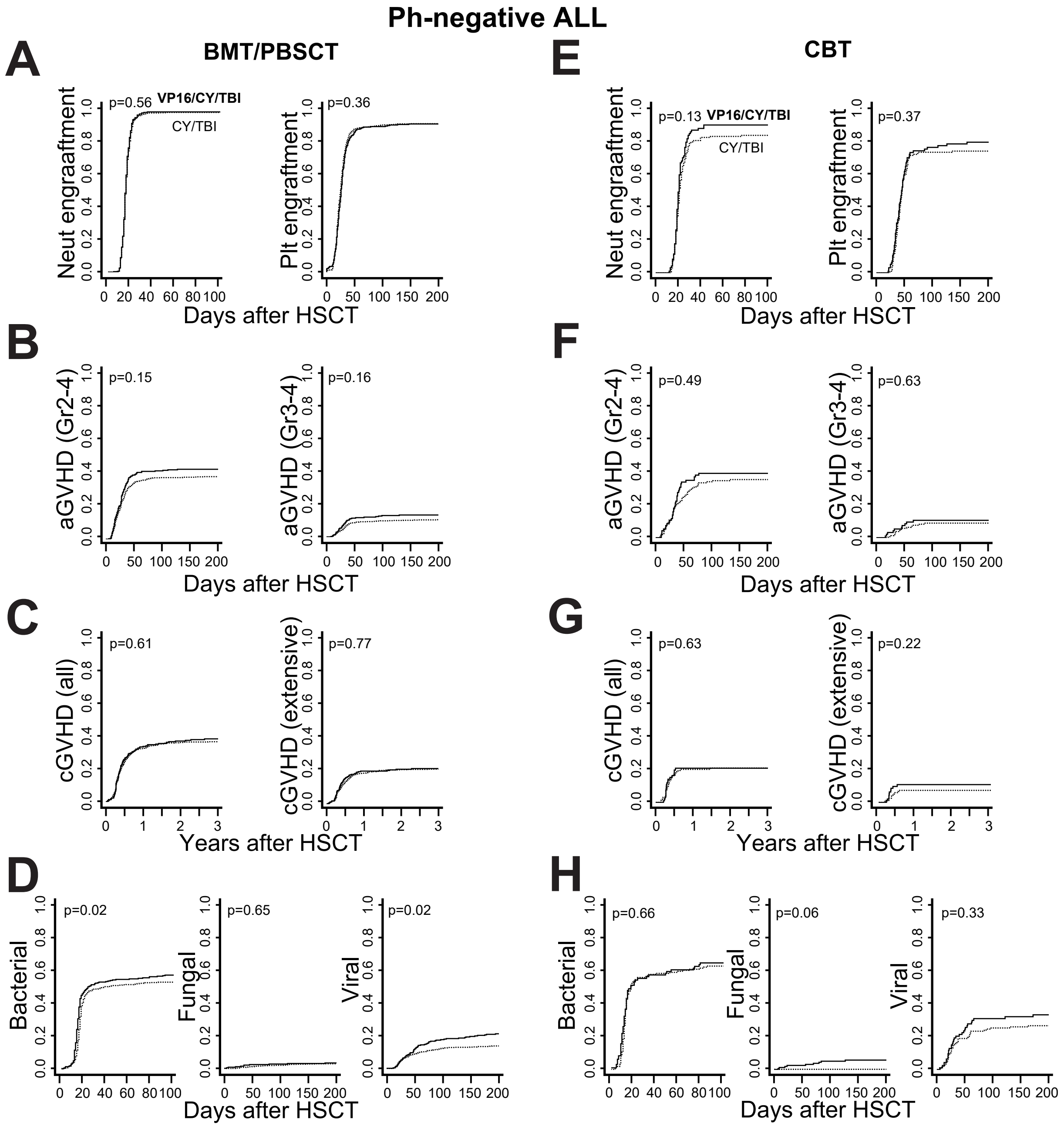

Supplement: Supplementary file 1 — Supplementary Material S1 [file HON-40-1041-s001.pdf]
